# Supplementary figures and images for: Probing the aggregated effects of purifying selection per individual on 1,380 medical phenotypes in the UK Biobank
Source: PLoS Genet. 2021 Jan 25;17(1):e1009337. doi: 10.1371/journal.pgen.1009337 (PMC7861521; doi:10.1371/journal.pgen.1009337)

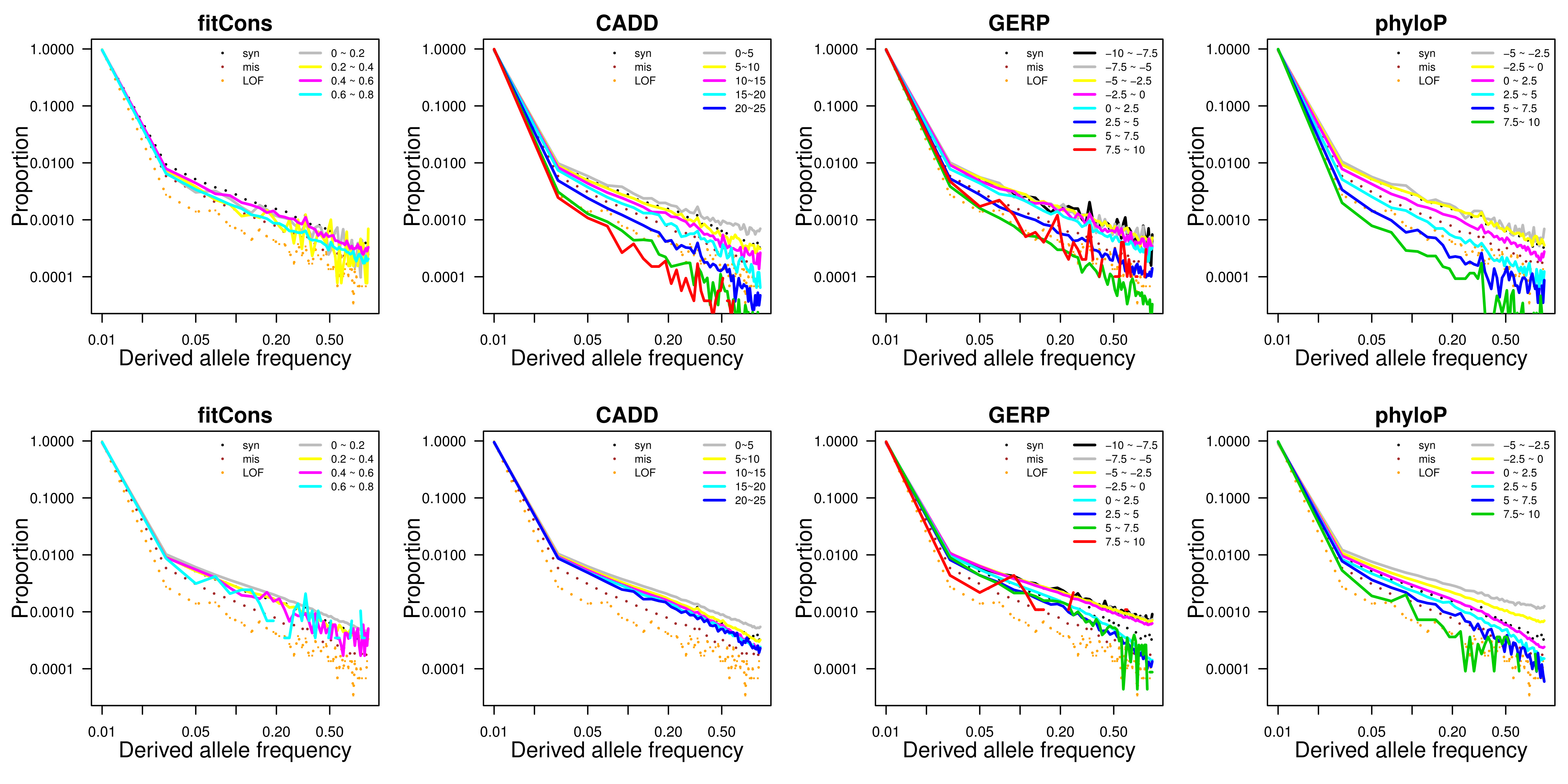

Supplement: S1 Fig — Top row: Derived allele frequency spectrum of coding variants. Bottom row: Derived allele frequency spectrum of non-coding variants. Each solid line represents derived allele frequency spectrum of polymorphic sites belonging to one score category and three dashed lines represent derived allele frequency spectra of three control categories: synonymous (syn), missense (mis), and loss of function (LOF) variants. (TIF) [file pgen.1009337.s001.tif]

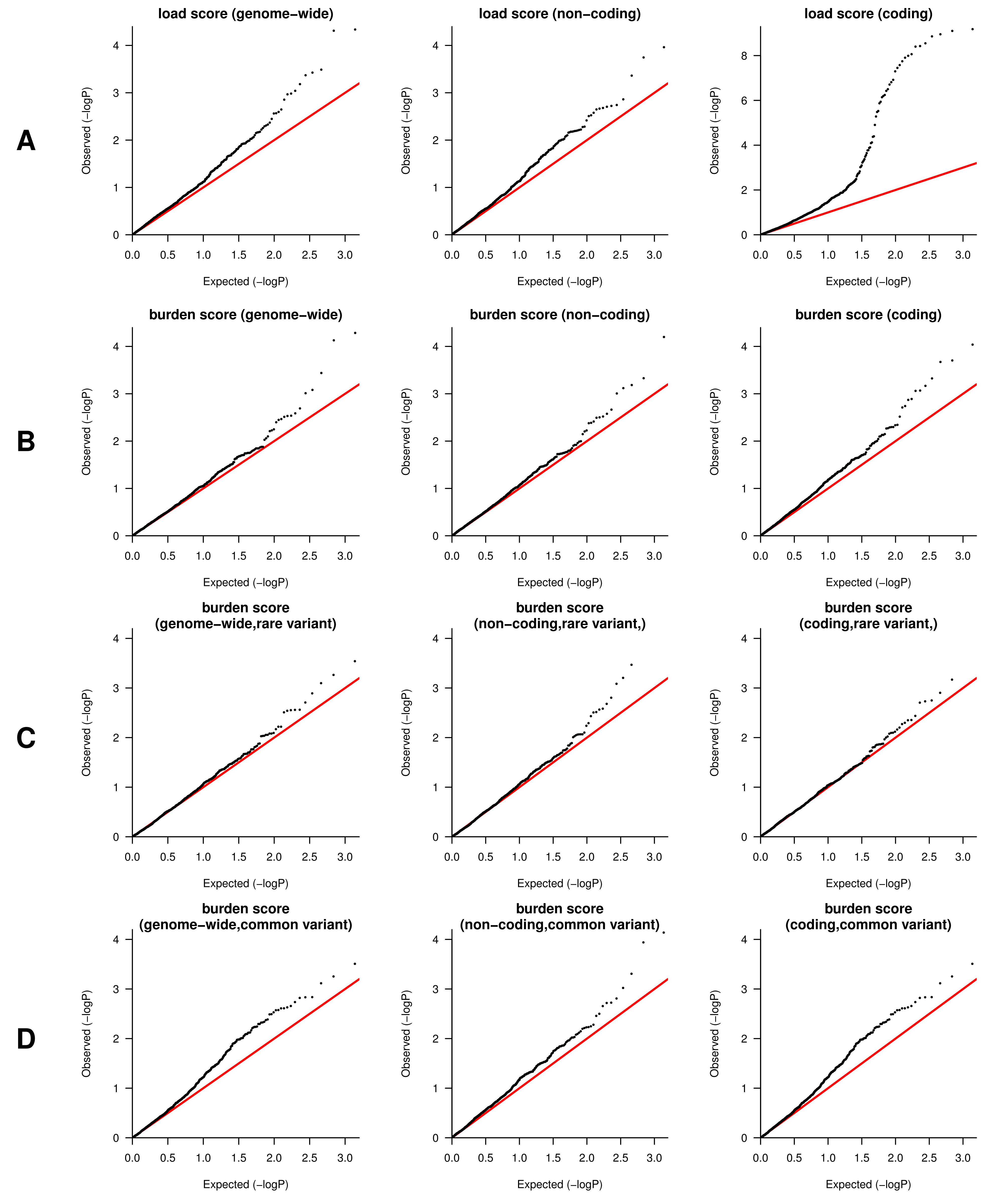

Supplement: S2 Fig — Quantile-quantile plot of -log10 p-values for the phenotypic association of A) load scores (weighted by phyloP); B) burden scores (unweighted); C) burden scores restricted to rare variants (DAF<5%); and D) burden scores restricted to common variants (5%< = DAF<70%). (TIF) [file pgen.1009337.s002.tif]

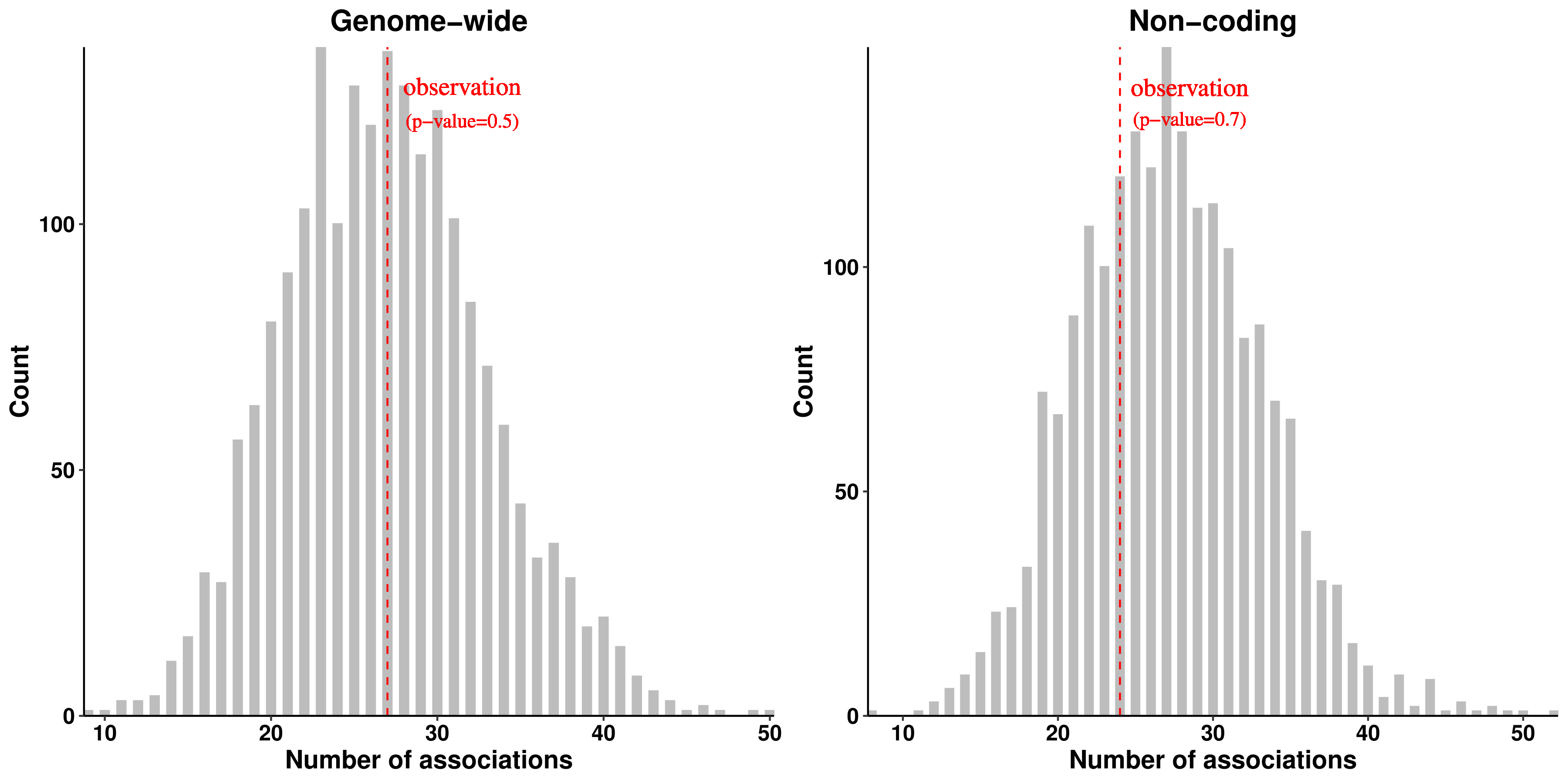

Supplement: S3 Fig — Null distribution of the number of clinical phenotypes weakly associated with genome-wide load score (left) and non-coding load score (right) was obtained from 2,000 permutations each. For each permutation, the load score was shuffled randomly among 335,161 samples and the number of associations on the x-axis was the count of phenotypes which yielded p-value < 0.05 in the association tests between the permuted load score and 539 phecodes. The red dashed lines indicates the observed number of clinical phenotypes nominally associated with genome-wide load score (n = 27, left) and non-coding load score (n = 24, right). (TIF) [file pgen.1009337.s003.tif]

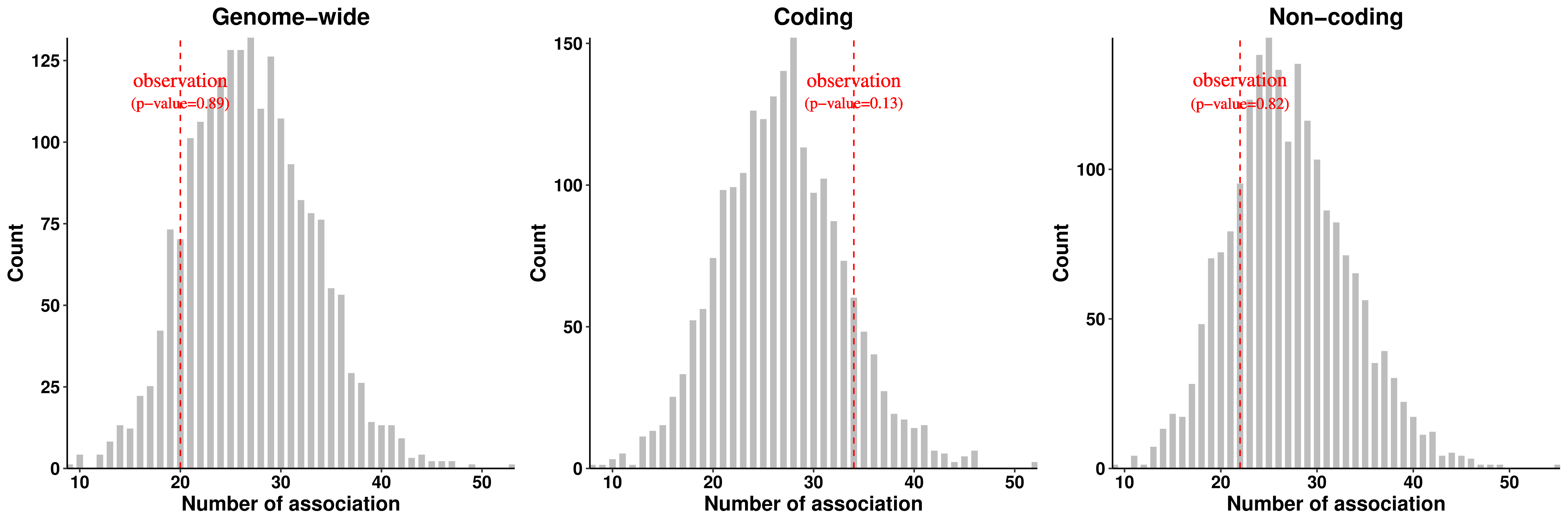

Supplement: S4 Fig — Null distributions of the number of clinical phenotypes weakly associated with burden scores were obtained using the same procedure to obtain the null distributions for load scores (Figs 3 and S3). The red dashed lines indicates the observed number of clinical phenotypes nominally associated with genome-wide burden score (n = 22, left), coding burden score (n = 20, middle), and non-coding load score (n = 20, right). (TIF) [file pgen.1009337.s004.tif]
