# Supplementary material for: Probing the aggregated effects of purifying selection per individual on 1,380 medical phenotypes in the UK Biobank
Source: PLoS Genet. 2021 Jan 25;17(1):e1009337. doi: 10.1371/journal.pgen.1009337 (PMC7861521; doi:10.1371/journal.pgen.1009337)
Supplement: S1 Table — This table shows the result of linear regression tests between the slopes of DAF spectra and score category ranks for each scoring method. The slope of a DAF spectrum is the slope of the best-fit linear regression line. Score categories from low to high are coded as an integer starting from 1. (DOCX) [file pgen.1009337.s005.docx]

**S1 Table.** Linear regression between slopes and score categories.

| **Score** | **beta** | **SE** | **P-value** |
| --- | --- | --- | --- |
| fitCons | 0.061 | 0.012 | 0.037 |
| GERP | 0.094 | 0.021 | 0.0064 |
| CADD | 0.13 | 0.026 | 0.0046 |
| phyloP | 0.15 | 0.014 | 0.00039 |

SE: standard error.
